# Association of Frailty and Suicide in Adults 65 Years and Older

**DOI:** 10.1093/geroni/igab046.2191

**Published:** 2021-12-17

**Authors:** Randall Kuffel, Ruth Morin, Kenneth Covinsky, John Boscardin, Yixia Li, Amy Byers

**Affiliations:** 1 San Francisco Veterans Affairs Health Care System, San Francisco, California, United States; 2 John D. Dingell VA Medical Center, Detroit, Michigan, United States; 3 University of California, San Francisco, San Francisco, California, United States; 4 San Francisco VA Health Care System, San Francisco, California, United States

## Abstract

Nearly 10,000 adults aged 65 years and older die by suicide in US annually. Although prior studies have linked individual diagnostic factors to late-life suicide risk, to our knowledge none have examined how accumulated health burden affects suicide risk. Such a metric could be studied utilizing a frailty index (FI). Our primary study objective was to determine the relationship of FI to risk of suicide. We examined a longitudinal cohort of 2,858,876 veterans 65 years and older from fiscal year 2012-2013 (baseline) through 12/31/2017, linking the VA’s suicide and mortality databases with medical record data. FI was defined by 31 variables, including morbidity, function, cognition, mood, sensory loss, chronic pain, and failure to thrive. We used Fine-Gray proportional hazards regression to examine time to suicide attempt (fatal and non-fatal). Our sample’s average age was 75 (SD 8), 88% White, 9% Black, and 98% male. Thirty-seven percent of veterans were non-frail, 30% were pre-frail, 17% mildly frail, 9% moderately frail, and 7% severely frail. Over the course of the study, 9,043 veterans had a documented suicide attempt with >60% fatal. After adjusting for race, gender, region, substance use disorder, and PTSD, risk of suicide attempt increased across frailty categories: Hazard ratios increased from 1.37 (95%CI: 1.30-1.45) for pre-frail individuals to 1.57 (1.43-1.72) for severely frail individuals. We found similar results after further adjustment for the Charlson Comorbidity Index, suggesting cumulative deficit FI may be a strong prognostic marker for risk of suicide in adults over 65; informing late-life suicide prevention efforts.

